# *Ixodes scapularis* density and *Borrelia burgdorferi* prevalence along a residential-woodland gradient in a region of emerging Lyme disease risk

**DOI:** 10.1038/s41598-024-64085-6

**Published:** 2024-06-07

**Authors:** James J. Logan, Anders Knudby, Patrick A. Leighton, Benoit Talbot, Roman McKay, Tim Ramsay, Justine I. Blanford, Nicholas H. Ogden, Manisha A. Kulkarni

**Affiliations:** 1 School of Epidemiology and Public Health, University of Ottawa, Ottawa, ON Canada; 2 Department of Geography, Environment and Geomatics, University of Ottawa, Ottawa, ON Canada; 3 Department of Pathology and Microbiology, Faculty of Veterinary Medicine, Université de Montréal, Saint-Hyacinthe, QC Canada; 4 Ottawa Hospital Research Institute, University of Ottawa, Ottawa, ON Canada; 5 Department of Earth Observation Science, Faculty of Geo-Information Science and Earth Observation, University of Twente, Enschede, The Netherlands; 6 Public Health Risk Sciences Division, National Microbiology Laboratory, Public Health Agency of Canada, Saint-Hyacinthe, QC Canada

**Keywords:** Ecological epidemiology, Urban ecology, Epidemiology

## Abstract

The environmental risk of Lyme disease, defined by the density of *Ixodes scapularis* ticks and their prevalence of *Borrelia burgdorferi* infection, is increasing across the Ottawa, Ontario region, making this a unique location to explore the factors associated with environmental risk along a residential-woodland gradient. In this study, we collected *I. scapularis* ticks and trapped *Peromyscus *spp. mice, tested both for tick-borne pathogens, and monitored the intensity of foraging activity by deer in residential, woodland, and residential-woodland interface zones of four neighbourhoods. We constructed mixed-effect models to test for site-specific characteristics associated with densities of questing nymphal and adult ticks and the infection prevalence of nymphal and adult ticks. Compared to residential zones, we found a strong increasing gradient in tick density from interface to woodland zones, with 4 and 15 times as many nymphal ticks, respectively. Infection prevalence of nymphs and adults together was 15 to 24 times greater in non-residential zone habitats. Ecological site characteristics, including soil moisture, leaf litter depth, and understory density, were associated with variations in nymphal density and their infection prevalence. Our results suggest that high environmental risk bordering residential areas poses a concern for human-tick encounters, highlighting the need for targeted disease prevention.

## Introduction

Lyme disease, a tick-borne illness in humans, which in North America is caused by infection with *Borrelia burgdorferi* sensu stricto (s.s.), is the most common vector-borne disease in Canada^1^. The number of confirmed Canadian cases of human Lyme disease was 2595 in 2021, an 18-fold increase above 2009 when it was designated a nationally notifiable disease^2^. The eastern provinces of Ontario, Québec, and Nova Scotia annually report most cases in Canada; together they accounted for more than 95% of the confirmed cases in 2021^1^. *Ixodes scapularis* (the blacklegged tick) is the vector of Lyme disease in these regions, where populations of this obligate parasite are rapidly expanding beyond its previously documented range and continue to establish in new areas^3–5^.

The environmental risk of Lyme disease is defined by the density of *I. scapularis* ticks and their prevalence of *B. burgdorferi* infection. In Ontario and Québec, forests—particularly those dominated by deciduous tree species—provide the ideal environmental conditions for tick survival through all developmental life stages^6^. The blacklegged tick thrives in wooded habitats that provide dense canopy cover, forest floor vegetation, and suitable densities of vertebrate hosts^7–9^. Immature stages of *I. scapularis* feed on rodents, including white-footed mice (*Peromyscus leucopus*), which are important reservoir hosts for *B. burgdorferi*^10^, or birds, while adult female ticks feed predominantly on white-tailed deer. Though the list of potential host species may be much longer, evidence in southern Ontario^11^ and southwestern Québec^12^ points to the relative importance of mice as competent reservoirs in this region. Understory vegetation and leaf litter in the forest microhabitat provide refuge for engorged and questing ticks that protect them from desiccation and extremes of heat and cold^13^. As annual temperatures increase at higher latitudes due to climate change, ticks carried northward by deer and migratory birds can establish new tick populations, resulting in Lyme disease risk emerging in new environments^14^. Past studies established the characteristics of the microhabitats associated with increased environmental risk for Lyme disease^7,15^, focusing on the ecological suitability of different woodland types for *I. scapularis* ticks and *B. burgdorferi* transmission. Yet the impact of forest fragmentation and urban encroachment into woodlands on local Lyme disease risk is not well understood.

Generally, Lyme disease risk has an inverse relationship with urban intensity, where highly developed areas contain little or no tick habitat. At the same time, urban expansion impacts the landscape around cities. Many Canadian metropolitan areas, including Ottawa, Ontario, experienced more rapid growth at the urban fringe and in the surrounding suburbs than through city centre intensification between 2016 and 2021^16^. These anthropogenic changes can manifest as the fragmentation of regional forests surrounding built-up land^17^. A common result is a mosaic of new developments mixed with numerous small forest patches or degraded forest complexes, which result in larger forest edge density and ecotonal habitat area.

Ecotonal habitat is linked to increased opportunities for interaction between humans and wildlife, including with blacklegged ticks^18^, and is associated with higher tick-borne disease risk^19–22^. Early studies exploring a connection between forest edge and tick abundance primarily focused on forest edge shared with herbaceous land cover and rarely with residential land^23–26^. Recent studies of peri-urban green space and urban parks also focus on the type of vegetative habitat present, reporting heterogeneity in the presence and density of ticks in public spaces^27–29^. Yet, in the United States and Canada, evidence from a variety of settings and regional scales supports the association of woodland-residential ecotones with increased risk of Lyme disease^30–33^. In the northeastern United States, it is thought that exposure to ticks is mainly peridomestic though the evidence for this focuses on study areas where both the pathogen and vector have long-established endemicity^34–39^. This regional understanding of Lyme disease risk in the local landscape does not necessarily translate to residential settings experiencing the emergence of blacklegged tick populations where there may be more heterogeneity in tick population densities and infection prevalence—thus also the environmental risk of Lyme disease—at fine spatial scales.

In their recent metanalysis, Fischhoff et al. identified that neighbourhood-level factors (e.g., woods adjacent to or within 500 m of a resident’s yard) are more predictive of tick bites and Lyme disease incidence than characteristics of the residential yards themselves^40^. Moon et al. similarly identified the need to consider peridomestic forest configuration at finer geographic scales (e.g., vicinity of residential yards compared to larger communities; cities compared to boroughs or townships) to better understand landscape risk factors associated with Lyme disease incidence^41^. Meanwhile, Keesing et al. studied discrete residential properties in New York and found a positive correlation between the proportion of forested property and tick abundance, yet no association with domestic indices of forest fragmentation^42^. Other studies demonstrate that white-footed mice avoid the residential forest edge in favour of woodland interior, suggesting that environmental control measures are best focused within peridomestic woodlands rather than in the habitat along the edge^43,44^.

These aspects of Lyme disease ecology are rarely analyzed simultaneously and at a fine scale where tick habitat and human activity intersect. Issues associated with Lyme disease risk in urban and peri-urban settings continue to attract significant research interest, with the focus ranging from property-level factors to urban greenspaces and in settings of emergent and established risk. Maupin et al.^35^ studied woods and unmaintained edges on residential properties in an endemic area of New York state, finding that nymphal and adult ticks were abundant in each of the habitats. In the United Kingdom, Hansford et al.^29^ found that the highest densities of infected nymphs were associated with edge habitat between woods and parks or grasslands in their evaluation of tick abundance in recreational urban greenspaces. In a region of New York with more recent emergence of Lyme disease risk, Piedmonte et al.^45^ measured tick abundance and pathogen prevalence at sixteen urban and rural sites, concluding that woodland habitat was an important driver of tick density compared to edge. Gregory et al.^46^ focused their analysis on residential property-level factors on Staten Island, another area of emergent risk in New York, and determined that this region possesses considerable risk in residential yards that appears driven by a property’s surrounding canopy cover. Through comparisons between forests, lawn, and gardens on individual residential properties in a different area of New York, Keesing et al.^42^ found forested areas on properties featured greater tick abundance than either lawns or gardens, but there was high variance in the abundance of ticks on lawns between neighbourhoods. Each of these projects measures the entomological hazard at several instances during the active tick season, at either sites distributed across a region or on specific properties of recruited participants. What is emphasized less in this extensive attention to urban risk is a “One Health” approach^47^—paying attention to both the involvement of wildlife in the enzootic transmission cycle of tick-borne pathogens and the integration of peri-urban residential communities with greenspace through trails and pathways that lead to more human activity in areas of risk. Dumas et al.^27^ evaluated associations of habitat and host species abundance with tick densities in southwestern Québec, though their intent was to demonstrate the heterogeneity of tick densities across a peri-urban natural park. In the Canadian context, where the recent and ongoing invasion of blacklegged tick populations means residents are unfamiliar with the local public health threat posed by Lyme disease, urban risk is understudied.

The objective of this study was to characterize the environmental risk for Lyme disease, at a fine local scale and in a region of ongoing blacklegged tick emergence, across an urban development gradient. We defined this gradient in terms of residential, woodland, and residential-woodland interface (i.e., the ecotonal area between the two, hereafter referred to as ‘interface’ for conciseness) zones. Guided by the *a priori * hypothesis that the local density and infection prevalence of nymphal and adult blacklegged ticks is higher in the woodland and ecotone transition relative to residential areas, we aimed to quantify these associations in peri-urban neighbourhoods. The nymphal life stage is the smallest tick instar that may carry *B. burgdorferi* infection and is challenging to detect on a human host, posing the most significant threat to public health^48^. Moreover, the detection of multiple life stages over consecutive years provides evidence of established tick populations, which pose greater environmental risk^49^. Our findings can help municipal and public health officials inform residents in areas of increased peridomestic risk of necessary Lyme disease prevention and intervention measures.

## Results

### Descriptive statistics

**Figure 1:**
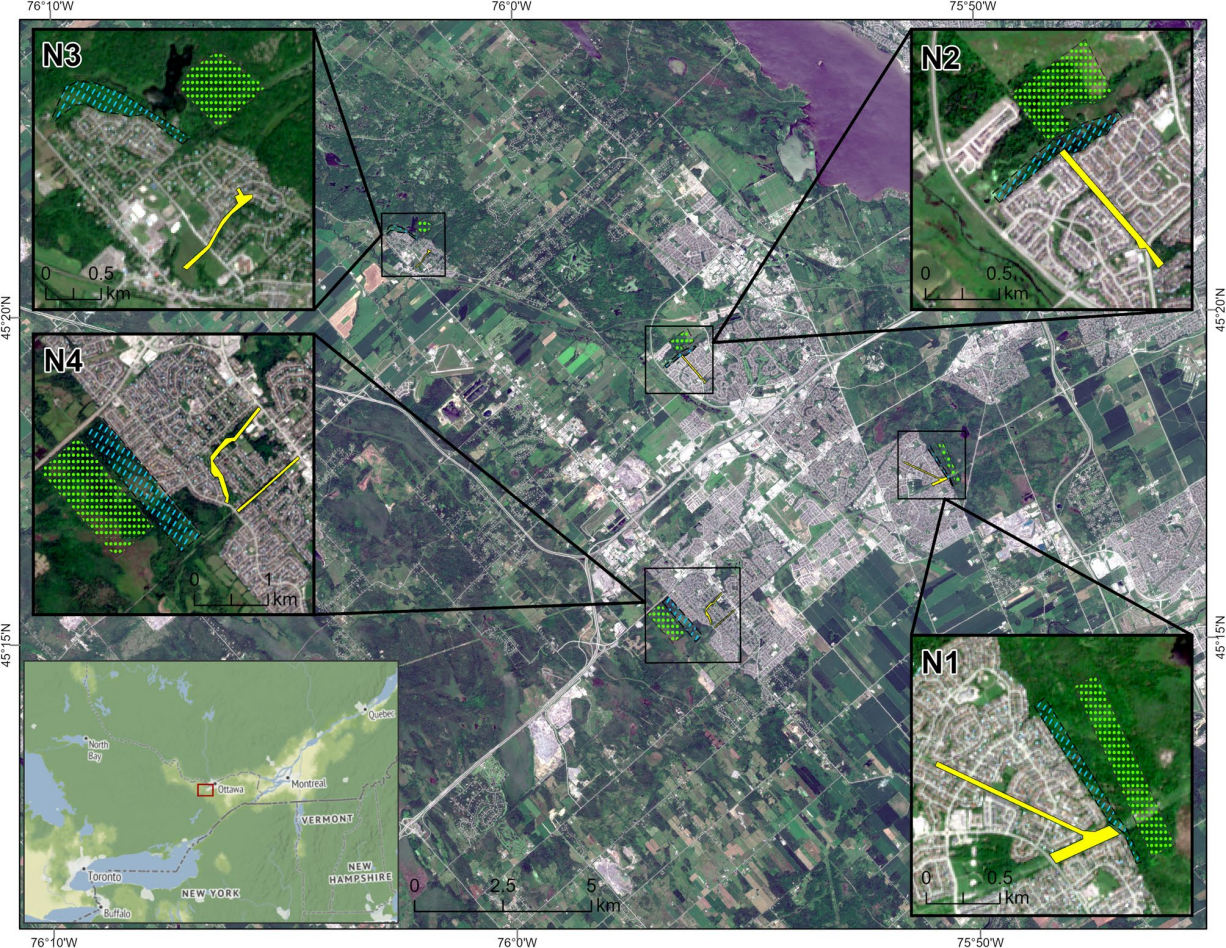
Location of residential (solid yellow fill), interface (light blue hatched-line fill), and woodland (bright green dot-hatched fill) sampling zones within each neighbourhood of western Ottawa: N1, N2, N3, and N4. The depicted zonal boundaries are approximations that include the locations of each drag sampling transect used during data collection, drawn to highlight their location and aid in visibility on this map. Basemap image is Copernicus Sentinel-2 data (2023), accessed and used in accordance with the regulations of the European Union. Context map base layer by Stamen Design, under CC BY 4.0, with data by OpenStreetMap, under the Open Database License (ODbL).

We completed 2736 quadrat observations from twelve grouped neighbourhood zones (three zones in each of N1-N4) in western Ottawa (Fig. 1) through nineteen rounds of tick drag sampling in 2020 and 2021. A total of 537 *I. scapularis* ticks were collected, of which 123 were nymphs, 300 were adults, and 114 were larvae (Table 1). More than half (54%) of the total ticks were collected in N2, three-quarters of which were from the woodland zone. The residential zone in N1 was the only area where we found no ticks of any stage over the entire study period. Also, no nymphal ticks were collected from the residential zone of N4. At least one adult tick was collected in the residential zone of all neighbourhoods except N1. Nymphs were present in the interface and woodland zones of all four neighbourhoods, with the total number ranging from 3 (N4) to 10 (N1) in interface zones and from 7 (N4) to 61 (N2) in woodlands. The difference in the number of adult ticks collected from the interface (22) and woodland (28) zones of N1 was small, while adult ticks collected from the other neighbourhoods’ interface zones were less than half of their woodland totals. Nymphs were collected most often between May and July, while adults were active between May and June and from September to the end of the study season in both years (see Supplementary Fig. S1 online). We collected larvae in all but one (N4) of the three study neighbourhoods. Though we observed none at residential sites, larval counts in N2 and N3 were higher in the interface (5 larvae collected in each) and woodlands (21 and 22, respectively). The interface zone of N1 proved to be the exception. In this zone, we found more than half (61) of all larvae collected—50 of these in one sampling effort.

**Table 1:** Total *Ixodes scapularis* ticks collected of each life stage with mean nymphal and all-stage *Borrelia burgdorferi*-infection prevalence and 95% confidence intervals (95% CI) by neighbourhood and zone.

Neighbourhood	Zone	Adults	Nymphs	Larvae	Total	Infected adults	Infected nymphs	Nymphal infection prevalence % (95% CI)	All-stage infection prevalence (95% CI)	*Haemaphysalis *spp. Larvae + Nymphs
N1	Residential	0	0	0	0	0	0	n/a (n/a)	n/a (n/a)	0
	Interface	22	10	61	93	8	0	0 (n/a)	25 (7, 37)	1
	Woodland	28	17	0	45	8	2	12 (0, 38)	22 (10, 37)	0
	N1 Total	50	27	61	138	16	2	7 (0, 21)	23 (13, 33)	1
N2	Residential	1	3	0	4	0	1	33 (0, 100)	25 (0, 100)	0
	Interface	64	6	5	75	21	3	50 (2, 100)	34 (22, 48)	1
	Woodland	129	61	21	211	50	14	23 (15, 37)	34 (22, 39)	0
	N2 Total	194	70	26	290	71	18	26 (17, 46)	34 (25, 39)	1
N3	Residential	1	1	0	2	0	1	100 (n/a)	50 (0, 100)	0
	Interface	7	4	5	16	3	1	25 (0, 100)	36 (1, 63)	0
	Woodland	15	11	22	48	5	1	9 (0, 31)	23 (6, 44)	2
	N3 Total	23	16	27	66	8	3	19 (0, 43)	28 (13, 43)	2
N4	Residential	2	0	0	2	0	0	n/a (n/a)	n/a (n/a)	0
	Interface	8	3	0	11	3	2	67 (0, 100)	45 (10, 81)	0
	Woodland	23	7	0	30	9	2	29 (0, 90)	37 (20, 63)	4
	N4 Total	33	10	0	43	12	4	40 (5, 95)	37 (24, 57)	4
All	Residential	4	4	0	8	0	2	50 (0, 100)	25 (0, 74)	0
	Interface	101	23	71	195	35	6	26 (7, 48)	33 (23, 41)	2
	Woodland	195	96	43	334	72	19	20 (12, 33)	31 (23, 36)	6
Overall		300	123	114	537	107	27	22 (16, 34)	32 (25, 36)	8

Of the 123 nymphs collected, 27 (21.9%) tested positive for *B. burgdorferi*, while 107 (35.7%) of the 300 adults collected were infected (Table 1). We collected infected nymphal ticks from all zones except for the residential of N1 and N4, and the interface of N1. The infection prevalence among all tested ticks from interface zones was equal to or greater than that of woodland zones. Across all four neighbourhoods and both sampling years, the nymphal infection prevalence was 22% while the combined adult-nymph infection prevalence was 32% (Table 1).

*Peromyscus* species mice were present in all neighbourhoods and zones. In total, we sampled 812 mice of the target species. *Peromyscus leucopus* (i.e., white-footed mice) comprised 69% of sampled mice across all neighbourhoods, with *P. maniculatus* (i.e., deer mice) representing 27%. *Peromyscus* spp. mice were collected in the greatest abundance in N1 and N4, with 40% and 35% of the total, respectively (Table 2). We collected most mice from the residential zone of each neighbourhood, except for N3 where we collected the most from the woodland zone (Table 2). The prevalence of *B. burgdorferi* infection among all sampled mice was 11% (Table 2), and 96% of infected mice were white-footed mice; only 4 sampled deer mice tested positive (Supplementary Table S1). Neighbourhood prevalence of *B. burgdorferi* infection among all mice ranged from 6% (N4) to 18% (N2). The residential zone of N1 was the only residential area with mice that tested positive for *B. burgdorferi*. Among *P. leucopus* mice, *B. burgdorferi* prevalence was between 17 and 32% in interface zones and 19% and 53% in woodland zones. Overall, 57% of the infected mice we sampled were collected in woodland zones (Supplementary Table S2). The mean deer foraging intensity was 0.5 events (i.e., photos of unique deer) per 100 trap-hours, with a range from 0.2 (N4) to 1.1 (N1). All zones of N1 exhibited the highest deer foraging intensity during the period our trail cameras were in place. This neighbourhood’s residential zone saw more unique events (1.8 per 100-trap hours) than any other study zone (Table 2).

**Table 2:** Total *Peromyscus* species mice collected, zonal abundance (mice per 100 trap-nights), *Borrelia burgdorferi* infection prevalence, and mean deer foraging intensity (events per 100 trap-hours) by neighbourhood and zone (‘R’: residential; ‘I’: interface; ‘W’: woodland).

Neighbourhood	Zone	*P. leucopus*			*P. maniculatus*			*P.* sp.^a^		Mean deer foraging intensity
		Total	Abundance Index	Infection prevalence	Total	Abundance Index	Infection prevalence	Total	Abundance Index	
N1	R	66	5.5	4.5	56	4.7	0.0	6	0.5	1.8
	I	65	5.4	18.5	26	2.2	3.8	3	0.3	0.9
	W	77	6.4	28.6	25	2.1	4.0	2	0.2	0.6
	Total	208	5.8	17.8	107	3.0	1.9	11	0.3	1.1
N2	R	32	2.7	0.0	9	0.8	0.0	0	0.0	0.0
	I	22	1.8	31.8	11	0.9	0.0	1	0.1	0.1
	W	21	1.8	52.4	6	0.5	0.0	0	0.0	0.4
	Total	75	2.1	24.0	26	0.7	0.0	1	0.03	0.2
N3	R	20	1.7	0.0	1	0.1	0.0	1	0.1	0.6
	I	27	2.3	18.5	10	0.8	0.0	0	0.0	0.2
	W	31	2.6	32.3	9	0.8	11.1	1	0.1	0.7
	Total	78	2.2	19.2	20	0.6	5.0	2	0.1	0.5
N4	R	109	9.1	0.0	29	2.4	0.0	12	1.0	0.0
	I	58	4.8	17.2	30	2.5	3.3	3	0.3	0.2
	W	36	3.0	19.4	5	0.4	0.0	2	0.2	0.3
	Total	203	5.6	8.4	64	1.8	1.6	17	0.5	0.2
Overall		564	3.9	15.4	217	1.5	1.8	31	0.2	0.5

^a^No *Peromyscus* sp. mice tested positive for *B. burgdorferi.*

We detected significant associations between all ecological characteristics and both sampling neighbourhoods and zones (p < 0.001). The most common tree species in all neighbourhoods were maples, though the difference in proportions of sampling quadrats dominated by maples compared to coniferous species was smaller in N3 and N4 (Table 3). Quadrats in interface and woodland zones were predominantly occupied by maple trees, while the dominant species among residential quadrats was more varied. Full canopy cover (69%) and understory density (41%) most characterized quadrats in woodland zones, though this was also true of N2 in general (Table 3). Shallow litter depth was most common in interface and woodland quadrats. Dry soil humidity characterized the majority of the neighbourhood and zonal quadrats.

**Table 3:** Proportions (%) of sampling quadrats with the recorded observation during June 2020 and 2021 site visits within each neighbourhood and each zone type, with descriptive statistics for continuous measurements.

Characteristic	Value	Neighbourhoods				Zones		
		N1 (n = 72)	N2 (n = 72)	N3 (n = 72)	N4 (n = 72)	Residential (n = 96)	Interface (n = 96)	Woodland (n = 96)
Dominant tree species	Ash	17	6	7	11	16	10	4
	Maple	56	50	39	28	32	55	42
	Cedar	0	7	7	14	2	8	10
	Coniferous	8	11	33	22	30	8	19
	Other decid	19	26	14	25	20	19	25
Understory density	Bare	47	0	24	42	51	23	10
	Sparse	22	17	29	44	19	31	34
	Dense	9	18	7	8	2	15	15
	Full	22	65	40	6	28	31	41
Litter layer depth	None	47	18	37	45	69	31	10
	Shallow	40	64	46	51	22	53	76
	Moderate	13	18	13	4	9	13	14
	Deep	0	0	4	0	0	3	0
Canopy cover mean (s.d.)		48.3 (47.9)	86.1 (26.1)	71.7 (40.7)	22.1 (33.2)	26.5 (37.5)	69.7 (41.0)	75.0 (39.5)
Litter layer depth mean (min–max)		1.11 (0–5)	1.30 (0–5)	1.20 (0–10)	0.87 (0–4)	0.55 (0–5)	1.35 (0–10)	1.46 (0–5)
Soil humidity mean (s.d.)		13.4 (5.7)	28.7 (20.9)	28.9 (23.0)	12.9 (4.8)	24.0 (23.5)	20.9 (14.9)	18.2 (12.6)
*P. leucopus* abundance (mice per 100 trap-nights) mean (s.d.)		5.8 (1.6)	2.1 (0.7)	2.2 (0.5)	5.6 (2.7)	4.7 (3.2)	3.6 (1.8)	3.4 (1.8)
*P. leucopus* *B. burgdorferi*-infection prevalence % (95% CI)		16.8 (7.6, 26.0)	28.4 (10.6, 46.2)	17.2 (5.4, 29.0)	12.2 (3.9, 20.5)	0.8 (0.0, 2.2)	21.3 (15.5, 27.1)	33.9 (24.5, 43.3)
Deer intensity (deer per 100 trap-hours) mean (s.d.)		1.1 (0.6)	0.2 (0.3)	0.5 (0.3)	0.2 (0.1)	0.6 (0.8)	0.4 (0.3)	0.5 (0.3)

### Statistical models

Compared to residential zones, nymphal density was between 4 (interface, 95% confidence interval (95% CI): 1.19–15.03, p < 0.001) and 15 (woodland, 95% CI: 4.40–49.85, p < 0.001) times greater when controlling for other factors (Table 4), presenting a strong gradient effect across zonal habitat types. The association between nymphal infection prevalence and woodland zones was similarly strong (odds ratio (OR): 13.62, 95% CI: 2.07–89.58, p = 0.007), though the relationship with interface zones was not statistically significant (Table 4). Analyses that assessed the density and infection prevalence of all potentially infectious ticks (i.e., nymphs and adults) again demonstrated a strong and significant zonal gradient effect (Table 4). The density of nymph and adult ticks was nearly 22 times greater in interface zones and over 31 times greater in woodland zones relative to those in residential zones. Moreover, the infection prevalence of all tested ticks was nearly 15 times greater in interface zones than in the residential (OR: 14.90, 95% CI: 3.15–61.15, p < 0.001) and just over 24 times greater in woodlands (OR: 24.23, 95% CI: 6.37–125.14, p < 0.001). Though a substantial overlap between the confidence intervals for interface and woodlands zones existed for all metrics, their estimates relative to residential zones were statistically significant (p < 0.001).

**Table 4:** Rate ratios (RR) for associations with *I. scapularis* nymphal and nymph and adult density and odds ratios (OR) for associations with *B. burgdorferi* infection prevalence of *I. scapularis* nymphs and ticks. All models are adjusted for sampling month, year, and repeated measurements in neighbourhoods. Estimates are reported with Wald 95% confidence intervals (95% CI) and *P* values.

Variable	Nymphal density		Nymphal infection prevalence		Nymph and adult density		Nymph and adult infection prevalence	
	RR (Wald 95% CI)	*P* value	OR (Wald 95% CI)	*P* value	RR (Wald 95% CI)	*P* value	OR (Wald 95% CI)	*P* value
Dominant tree species (ref: ash)								
Maple	1.45 (0.29, 7.21)	0.654	0.46 (0.04, 4.79)	0.515	1.55 (0.79, 3.02)	0.199	1.11 (0.38, 2.91)	0.842
Cedar	2.52 (0.42, 15.27)	0.314	0.31 (0.02, 5.50)	0.425	1.29 (0.58, 2.88)	0.531	1.04 (0.26, 2.89)	0.948
Other coniferous	0.98 (0.15, 6.43)	0.983	0.00 (n/a)	0.973	1.57 (0.70, 3.54)	0.276	0.22 (0.03, 0.96)	0.069
Other deciduous	0.91 (0.17, 4.82)	0.910	0.08 (0.01, 1.27)	0.073	1.09 (0.54, 2.20)	0.801	0.75 (0.27, 2.25)	0.602
Canopy cover (+ 10%)	1.04 (0.92, 1.18)	0.523	1.08 (0.87, 1.34)	0.494	0.99 (0.93, 1.06)	0.737	0.96 (0.88, 1.07)	0.452
Understory density (ref: bare)								
Sparse	0.63 (0.19, 2.06)	0.447	0.03 (0.00, 0.24)	0.001	1.36 (0.73, 2.51)	0.332	0.79 (0.33, 2.45)	0.635
Dense	0.15 (0.03, 0.76)	0.022	0.02 (0.00, 0.42)	0.011	1.32 (0.63, 2.74)	0.464	0.79 (0.30, 2.75)	0.676
Full	0.91 (0.26, 3.15)	0.876	0.13 (0.02, 0.91)	0.040	2.49 (1.30, 4.78)	0.006	1.17 (0.47, 3.94)	0.772
Litter layer depth (ref: none)								
Shallow	3.80 (1.22, 11.80)	0.021	15.49 (1.70, 141.12)	0.015	1.65 (1.00, 2.70)	0.049	2.54 (0.95, 4.80)	0.026
Moderate	2.90 (0.85, 9.93)	0.090	6.56 (0.60, 71.98)	0.124	0.92 (0.49, 1.70)	0.786	1.55 (0.39, 3.08)	0.392
Soil moisture (+ 10%)	1.21 (1.01, 1.45)	0.036	1.48 (1.13, 1.93)	0.004	1.02 (0.91, 1.14)	0.761	1.15 (0.95, 1.30)	0.060
* P. leucopus* abundance index			0.85 (0.62, 1.17)	0.329			0.84 (0.63, 1.04)	0.165
Mean deer foraging intensity (× 100)	2.21 (0.97, 5.03)	0.060	0.72 (0.13, 4.04)	0.711	1.53 (0.94, 2.49)	0.087	1.67 (0.58, 2.77)	0.192
Zone type (ref: residential)								
Interface	4.24 (1.19, 15.03)	< 0.001	1.69 (0.25, 11.45)	0.593	21.79 (9.94, 47.77)	< 0.001	14.90 (3.15, 61.15)	< 0.001
Woodland	14.81 (4.40, 49.85)	< 0.001	13.62 (2.07, 89.58)	0.007	31.33 (14.25, 68.86)	< 0.001	24.23 (6.37, 125.14)	< 0.001

In both models of nymphal tick outcomes, we identified associations with several aspects of the forest microhabitats. Shallow litter depth (i.e., less than 2 cm) compared to no leaf litter was associated with nearly 4 times greater density of nymphs (95% CI: 1.22–11.80) and a more than 15-fold higher nymphal infection prevalence (95% CI: 1.70–141.12). A ten-percent higher soil moisture above average was also associated with a 20% higher nymphal density (rate ratio (RR): 1.21, 95% CI: 1.01–1.45) and a nearly 50% greater infection prevalence among nymphs (OR: 1.48, 95% CI: 1.13–1.93). We noted a strongly negative relationship between nymph outcomes and understory density, particularly with dense understory relative to bare (nymphal density RR: 0.15, 95% CI: 0.03–0.76; infection prevalence OR: 0.02, 95% CI: 0.00–0.42). However, a full understory was associated with a 2.5 times greater density of nymphs and adults together compared to a bare understory (RR: 2.49, 95% CI: 1.30–4.78).

**Figure 2:**
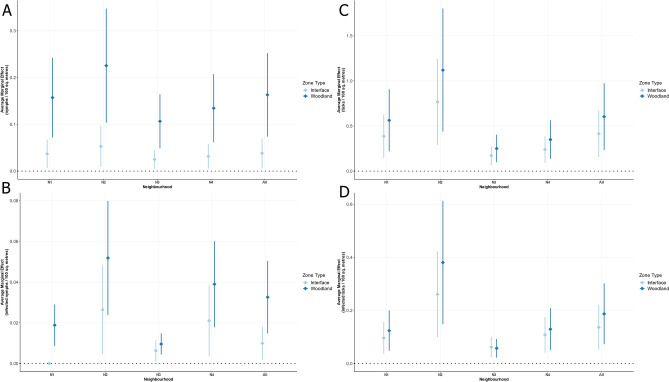
Average marginal effect on nymphal density (**A**), density of infected nymphs (**B**), tick (nymph and adult) density (**C**), and density of infected adults and nymphs (**D**) for interface and woodland zones compared to residential, with all other covariates at their mean value. Plots include the zonal effect within each neighbourhood (N1–N4) as well as independent of neighbourhood. The density of infected nymphs (**B**) and infected adults and nymphs combined (**D**) is determined by the product of the predicted density and the observed infection prevalence reported in Table 1.

Tick densities predicted by the average marginal effects demonstrate zonal and neighbourhood associations with the environmental risk of Lyme disease when site-specific and ecological characteristics are held at their mean or referent value (Fig. 2; Supplementary Table S3). Heterogeneous tick densities exist between the studied neighbourhoods; zones in N1 and N2 exhibited greater densities compared to those in N3 and N4 (Fig. 2A,C). Overall, predicted nymphal density was 0.05 per 100 m^2^ in interface zones compared to 0.17 per 100 m^2^ in woodlands. This zonal gradient on nymphal density was most evident in N2 (0.07 per 100 m^2^ and 0.24 per 100 m^2^ in the interface and woodland zones, respectively). The same gradient effect across zonal habitat types was present in predictions of the density of all potentially infectious ticks (i.e., nymphs and adults), both within and independent of neighbourhoods. Diagnostic plots of residuals indicated good model fits given the chosen response distributions, covariates adequately explaining the outcomes, and the absence of influential outliers (Supplementary Figs. S2–S5).

## Discussion

Our study provides a fine-scale examination of Lyme disease environmental risk along a landscape gradient, demonstrating elevated risk in interface and woodland zones relative to residential paths and trails. The density and infection prevalence of nymphal ticks in these ecotonal zones were higher than those found in residential zones, highlighting the significant role they hold in the Lyme disease risk present in neighbourhoods featuring residential areas with integrated greenspace. Studies of habitat types within urban woodland parks of England found the highest densities of *I. ricinus* nymphal ticks in woodland edge sites, with evidence of established populations in grassland, woodland, and woodland edge habitats of urban and peri-urban greenspaces^28,29^. Conversely, in New York state, Horobik et al. found that densities of *I. scapularis* ticks and infected ticks were both higher in forests than at forest-field edge, concluding that edges do not pose a higher risk than the forests themselves^50^. However, each of these studies examined the entomological risk present at the edge of forests with open grassland. We examined the forest edge that specifically interfaces with the boundary of residential properties. One hypothesized mechanism underlying the effect that human-driven landscape change has on Lyme disease risk is termed “ecological release”, wherein populations of a species increase due to changes that remove constraints limiting their expansion. A relevant example of this is the creation of new forest edges or clearings—specifically when these border human residential areas that provide new resources and nesting opportunities favoured by, for example, white-footed mice^22^. By accounting for possible differences in the abundance of white-footed mice in the sampled zones, which is believed to be the primary reservoir in this region^11,12^, as well as characteristics of the microhabitat known to impact blacklegged tick survival, we shed light on the role of interface zones on Lyme disease risk.

Direct comparison of total tick density between the unique zones and neighbourhoods indicates heterogeneity at this scale. Densities of nymphs and all potentially infectious ticks in N2 reflected previous estimates for the Ottawa municipal region^51^. The densities identified in each of the three remaining study neighbourhoods were lower. Still, despite a small sample size of infected nymphal ticks from these three neighbourhoods, we detected an infection prevalence that also supports estimates reported previously for this region, indicating the presence of Lyme disease environmental risk^51^. It is notable, however, that the prevalence of infection among the collected nymphs was consistently higher in interface zones compared to woodlands, apart from N1 where no infected nymphs were sampled. We also note that, while evaluating the density of nymph and adult ticks together is common, doing so carries potential issues in the assessment of infection rates given that prevalence in adult ticks is typically greater, having potentially fed twice on reservoir hosts. Still, the zonal differences we detected underscore the importance of considering the ecological relationship with reservoir hosts along the environmental gradient we examined.

Our results from sampled *Peromyscus*-species mice indicated higher abundance in residential zones while *B. burgdorferi* prevalence was highest in woodland habitats. This supports previous evidence demonstrating greater white-footed mouse abundance at sites with less forested area within a 500-m radius yet lower *B. burgdorferi* prevalence than their within-woodland kin^44^. Together, these results are demonstrative of the differences in scale-based associations of landscape edge effects. Though the difference in nymphal infection prevalence we measured for the interface zone was not statistically significant, the findings of Piedmonte et al. corroborate a similarity in prevalence among all potentially infectious ticks through their exploration of multi-scale habitat effects on tick-borne pathogen prevalence^45^. Continued surveillance in residential-woodland ecotonal habitats, where the potential for human-tick encounters is high, will improve our understanding of this relationship. And, while the *B. burgdorferi* prevalence of white-footed mice observed in our data appears to support its role in enzootic transmission within this region, the involvement of other potential host species remains understudied. Additional rigorous evaluation of host community composition would help to more fully characterize the transmission dynamics between these zonal settings^52,53^.

Several limitations of this study should be considered along with the results described above. Weak relationships between some ecological observations and the evaluated outcomes may be due to the generalization of each 50 m^2^ sampling area to discrete observations. We cannot dismiss the possibility that environmental measurements were subject to observer bias that resulted in misclassification. While it is a strength of this study design to investigate microhabitat characteristics of the sampling area where ticks were collected, the precision of effects for ecological characteristics could be further improved by quantifying the same measures. For example, obtaining imagery from drones flown along sampling transects and using machine learning image processing methods to inventory and calculate distributions of tree species would be a more efficient and less costly approach to quantify these areas of risk and guide field sampling. In terms of zonal factors, the final models we evaluated were adjusted for the involvement of white-footed mice and deer in the outcome measures, but we did not account for the structure or diversity of the broader vertebrate host community; we were not able to focus on other small or medium-sized mammal species. While we adjusted for study neighbourhood in our models, differences in landscape configuration of zones within neighbourhoods that were not measured may impact the results and should be an area for further research. We also determined tick density and infection prevalence using drag sampling which is less resource-intensive than the collection of ticks from small mammal hosts but may be less efficient^54–56^, though its effectiveness as a method to identify *I. scapularis* populations in this region has been shown^57^. We followed rigorous standardized protocols for drag sampling, but stage-differential or non-detection of ticks (false negatives) are a recognized limitation of this method. *I. scapularis* adults are generally easier to collect by drag sampling as some types of terrain more easily dislodge nymphal ticks (N.H. Ogden, personal communication, February 2, 2024). Finally, the prevalence calculations for some of the neighbourhood zones were based on small totals of collected nymphs and adults, which can result in wider confidence intervals due to uncertainty in the estimates^58^. Future research should continue active surveillance in these and additional neighbourhoods and other Canadian regions with emerging tick populations to further validate these estimates and their relationships.

Urban expansion involves the encroachment of residential developments into natural areas. As suburbs and peri-urban regions continue to change through this practice, it is crucial to understand how new ecotones contribute to local Lyme disease risk. Several past studies connect the quantity of wooded space in and around the residential yard to the increased risk of encounters with host-seeking ticks^35,46^. Yet these findings focus on Lyme disease risk where established residential properties border wooded areas and do not assess where new landscape change integrates existing forest with new residences, trails and pathways. Ours is among the first to quantify the effect of land cover types in different stages of urban development on local tickborne disease risk, specifically using a “One Health” approach across the residential-to-woodland ecotonal gradient. In this study, we focused on the fine-scale localization of Lyme disease risk within neighbourhoods of a region experiencing the rapid emergence of blacklegged tick populations. By simultaneously modelling the local impact of wildlife host availability, microhabitat characteristics, and stage of land development on the density and infection status of *I. scapularis* ticks, we demonstrated that the environmental hazard of tick-borne diseases is significant at the intersection of urban greenspace and residential properties. These results may help to inform urban planning guidelines regarding the selection and development of sites through the characterization of environmental Lyme disease risk prior to the development of new residential neighbourhoods. Continued surveillance, informing targeted control measures^59^, is necessary to ensure that local Lyme disease risk is minimized as new ecotonal “interface” areas allow human tick encounter opportunities. Finally, these effects should be investigated in other settings experiencing blacklegged tick emergence, to better understand the environmental characteristics of Lyme disease hazard and incorporate intervention and control measures into expansion plans.

## Methods

### Study location

Four neighbourhoods across the rural and suburban areas of western Ottawa (Fig. 1) were selected to assess tick populations, characterize the environmental factors and estimate the abundance of species involved in the *B. burgdorferi* transmission cycle (i.e., *Peromyscus-*species mice and white-tailed deer). Guided by the results of recent active tick surveillance that established the presence of black-legged tick populations^51^, we engaged in partnership and consultation with the National Capital Commission, Ottawa Public Health, and the City of Ottawa’s Planning Division to arrive at an informed selection of these sites. To identify characteristics related to varying degrees of proximity to urban and residential development, we subdivided each neighbourhood into three sampling zones: residential, woodland, and interface (Fig. 1). Residential zones consisted of pathways and trails within neighbourhoods between the back yards of residential properties, while woodland zones were defined as forested areas adjacent to the developed areas of neighbourhoods and connected to residential areas by trail networks. Interface zones consisted of trails and wooded edges immediately bordering residential properties, such that homes or fences remained visible from just inside the tree line.

### Tick drag sampling

Our study area included 36 sites evenly divided across the four neighbourhoods (N1–N4), with three sampling zones in each neighbourhood (i.e., residential, woodland, and interface), and three sites in each sampling zone.

Field collectors experienced in collecting ticks by drag sampling visited each site monthly from May through October 2020 and biweekly across the same period in 2021. At each site, team members followed a standard tick-dragging sampling protocol, wherein a 1-m white flannel sheet is dragged along the ground to collect questing ticks^60^. The starting location for each tick drag transect was selected based on observations from preliminary site visits that a continuous linear drag remained in the same zone type without interruption and allowed for measurement from three consecutive sites across both sampling years. Field collectors stopped every 25 m to check and remove ticks from the flannel and record the geographical coordinates of the sampling location using a Garmin eTrex 20 GPS, covering 200 m. In total, the area dragged per sampling visit was 600 m^2^ in each zone and 1800 m^2^ in each neighbourhood. All collected ticks were returned to the lab in specimen tubes, where species and stage were identified by microscope according to standard taxonomic keys^61–63^.

### Ecological characteristics

To ascertain any distinguishing characteristics that might influence the suitability of distinct locations for tick survival, we examined the fine-scale aspects of the microhabitat at each surveillance site. Field collectors recorded site-specific ecological measurements (Table 5) derived from several studies of blacklegged tick abundance at every second sampling location (50 m) in June of both study years. Mid-season values for all variables were used to capture and adjust for general ecological differences across all sites in our analyses.

**Table 5:** Ecological observations and measurements recorded at sampling locations, with description and rationale for consideration.

Variable	Units/categories	Rationale
Dominant tree species	Ash (reference) Maple Cedar Other coniferous Other deciduous	Deciduous trees provide heavier leaf litter and have been associated with higher levels of risk. Coniferous trees have been shown to discourage tick establishment due to the absence of leaf litter in some regions^9,74^
Canopy cover	%	A dense or closed canopy cover can protect from weather extremes (cold, wet) and provide more leaf litter^11,15^
Understory density	Bare (none present; reference) Sparse (visible but no significant coverage) Dense (thick, difficult to traverse) Full (healthy, covering most of the forest floor)	Habitats with a dense understory may provide better refuges for ticks from extreme weather and a grassy substrate on which to quest^9,66,75–77^
Litter layer depth	None (0 cm; reference) Shallow (< 2 cm) Moderate to deep (3 to 10 cm)	Leaf litter provides a refuge from cold in winter and keeps the microhabitat humid, which contributes to tick survival by protecting them from desiccation^9,11,15,66,77^
Soil moisture	%	In microhabitats, soil moisture is directly related to the relative humidity, which contributes to the ability of ticks to survive^9,66,74,77^

We identified all categorical variables according to previously established classification keys^9,64–66^. Field researchers also measured the percentage of canopy cover overhead at sampling locations using a spherical densiometer, according to established field protocols^67^. The same study personnel measured litter layer depth with a small ruler perpendicular to the ground, rounding to the nearest centimetre, and measured soil moisture in percentage by inserting a moisture meter probe approximately 4–6 cm into the soil. In analyses, we also treated litter depth as a categorical variable with the thresholds used by Talbot et al.^9^: none, shallow (< 2 cm), or moderate to deep (3–10 cm).

### Small mammal sampling

Our trapping effort was based on a target sample size of up to 50 individuals per species (i.e., *Peromyscus leucopus,* or white-footed mice, and *Peromyscus maniculatus*, or deer mice) per zone each year, assuming 1 mouse for approximately every 10 trap-nights with additional trap-nights to account for trap failures. Trapping sessions took place in two rounds of two consecutive nights in each neighbourhood zone during the peak period of mouse activity (June to August). Ethical approval for small-mammal research was obtained from the University of Ottawa Animal Care Committee (permit ME-3079). All animal trapping and handling methods were performed in accordance with the Canadian Council on Animal Care’s guidelines involving wildlife^68^.

During the two-night sampling period in each neighbourhood zone, field collectors laid 150 Sherman traps, baited with an apple cube, a pinch of oats, and a cotton round, on both sides of the trail. Team members ensured trap placement in two parallel lines 10 m apart, with 5–10 m of distance between each trap in the same line. The exact distance between traps varied depending on the habitat and availability of forested space in each location. Traps were set each evening and checked the following morning. Therefore, our sampling effort was 600 trap-nights per neighbourhood zone in each sampling year, totalling 7200 trap-nights across all neighbourhoods.

Small mammals captured during the night were briefly removed from traps into a cotton bag to safely measure their weight before removing them for inspection. For all trapped animals of the target species, sex, reproductive condition, length and weight were recorded, attached ticks were collected, and one ear punch biopsy was taken from each ear^69^ before the animal was released. Individuals for which the species could not be identified based on field guides were referred to as “*P.* sp.” in field records^70^. For any non-target animals trapped (e.g., chipmunks, voles), we identified the species, collected any attached ticks and released them. We did not sample these diurnal species as the capture methods we employed are tailored to nocturnal animals.

### Pathogen testing

We tested all biopsy ear punches, adult, and nymphal ticks for *Borrelia burgdorferi* s.s. using quantitative polymerase chain reaction (qPCR) assays according to previously published protocols^71,72^. In short, we extracted total genomic DNA using the QIAamp mini kit (QIAGEN, Mississauga, ON, Canada). With this, we identified *Borrelia* species using a duplex qPCR assay targeting the 23S ribosomal RNA. We further confirmed the presence of *Borrelia burgdorferi* s.s. by targeting the ospA gene. We performed amplifications with the BioRad CFX96 Real-Time PCR Detection System and sample analysis with CFX Maestro Software version 2.3 (https://www.bio-rad.com/en-ca/product/cfx-maestro-software-for-cfx-real-time-pcr-instruments, Bio-Rad Laboratories, Hercules, CA, USA).

### Deer density

As a proxy of white-tailed deer density in our models of tick outcomes, we calculated deer foraging intensity in each zone as captured by trail cameras. We installed one trail camera at each neighbourhood zone (12 total locations) at the end of June each year to collect images of large mammals active in the area. Every two weeks until the middle of October, field collectors relocated each camera 200 m along the tick drag sampling site, resulting in 8 camera trap sites per neighbourhood zone. We filtered all collected photographs to select only images of white-tailed deer and totalled the number of use events such that a unique deer captured by the camera counted as one event. To avoid overestimation due to double-counting, photographs with deer captured within a 10-min interval of the previous capture were considered a single event to allow for the individual’s activity to cross in and out of the camera’s detection zone. We estimated the foraging intensity at each camera location by dividing the number of events by the total number of camera-hours at the site, as per previously documented calculation methods^73^. We then averaged this number across all twelve installation locations for the camera to arrive at a “foraging intensity” estimate for deer activity in each neighbourhood zone.

### Statistical analysis

We linked ecological data to the total number of *I. scapularis* ticks of each stage from the current and preceding sampling locations. This resulted in 50-m-long rectangular quadrats as the unit of analysis, with four quadrats in each of the three sampling sites of all neighbourhood zones (144 in total). Each quadrat was dragged for questing ticks 19 times across the entire study period. Prior to analysis, the most dominant tree species was reclassified as one of ‘ash,’ ‘maple,’ ‘cedar,’ ‘other coniferous,’ or ‘other deciduous’ due to low numbers of observations for some deciduous and coniferous tree species as predominant among the sampling quadrats and to focus on effects of species with potential associations identified by Talbot et al.^9^. In all analyses, we mean-centred continuous variables: the proportion of canopy cover, soil moisture, and mean deer foraging intensity.

We performed all statistical analyses using the ‘lme4’ package in R version 4.1.3. Given the repeated transect measures performed within study neighbourhoods, we built generalized linear mixed models (GLMMs) with a random intercept for neighbourhoods to account for between-neighbourhood differences in the density and infection prevalence of ticks, as well as unmeasured differences in the configuration of neighbourhoods affecting relative distance between zones within neighbourhoods. In each model, we also forced month as an ordinal variable (5 to 10, May to October) to account for seasonal variation in peak activity of different tick life stages, and we used year as a factor with two levels (2020 and 2021) as an adjustment for interannual variation in tick abundance. To determine the most appropriate statistical distribution for our count data, we used the ‘glmer’ function to evaluate the association of our main predictor of interest, zone type, with tick densities in Poisson GLMMs. We checked these models for excess observations of zero ticks with the ‘check_overdispersion’ function from the ‘performance’ package. As overdispersion was detected, we proceeded with a negative binomial (NB) GLMM via the ‘glmer.nb’ function and checked the dispersion statistic again. Since the NB distribution adjusted for the overdispersion sufficiently, we deemed a zero-inflated process unnecessary. For models assessing associations with the presence of infected nymphs and adults, we used a Binomial GLMM with the ‘glmer’ function to estimate *B. burgdorferi* prevalence across our study sites.

For each outcome measure, we tested multivariable conceptual models conceived a priori. Negative binomial GLMMs exploring associations with the densities of nymphal and adult ticks included categorical variables (see Table 5 for ecological category definitions) for the dominant tree species, depth of leaf litter, understory density, and zone type, as well as continuous measurements for soil moisture, mean deer foraging intensity, and canopy cover. In Binomial GLMMs exploring the *B. burgdorferi* infection prevalence of nymphs and adults, we also included continuous predictors for the abundance and the *B. burgdorferi* infection prevalence of *P. leucopus* mice. We initially considered including predictors for *P. maniculatus* mice but omitted them due to their lower relative abundance between neighbourhoods and the near-total absence of *B. burgdorferi* infection among the sampled deer mice. A check of variance inflation factors among our model terms with the ‘performance’ package revealed collinearity between *P. leucopus* infection prevalence and zone type in both Binomial GLMMs. Thus, *B. burgdorferi* infection prevalence in white-footed mice was removed from the final models. For both count models, we calculated predicted tick densities based on the average marginal effects for neighbourhoods and zones with all covariates held at their mean values using the ‘margins’ package. We also performed residuals diagnostics with the ‘DHARMa’ package for hierarchical models.

### Supplementary Information

Supplementary Information.

## Data Availability

Ecological and observational surveillance data that support the findings of this study can be accessed on the University of Ottawa Dataverse: Logan, James Joseph; McKay, Roman; Kulkarni, Manisha, 2024, “Supporting Data for UPTick Project Active Surveillance, 2020 to 2021”, 10.5683/SP3/MCFVTB, Borealis, V1.
