# Supplementary material for: Ixodes scapularis density and Borrelia burgdorferi prevalence along a residential-woodland gradient in a region of emerging Lyme disease risk
Source: Sci Rep. 2024 Jun 7;14:13107. doi: 10.1038/s41598-024-64085-6 (PMC11161484; doi:10.1038/s41598-024-64085-6)

Supplementary Table S1. *Borrelia burgdorferi* infection status of mice by species

	Infected n (%)	Uninfected n (%)	Total
P. leucopus	87 (96)	477 (66)	564
P. maniculatus	4 (4)	213 (30)	217
Other *P. sp.*	0 (0)	31 (4)	31
Total	91	721	812

Supplementary Table S2. *Borrelia burgdorferi*-infection status of mice by zone and neighbourhood.

	Residential		Interface		Woodland		Neighbourhood Total (%)
	Positive	Negative	Positive	Negative	Positive	Negative	
Neighbourhood 1	3	125	13	81	23	81	39 (11.9)
Neighbourhood 2	0	41	7	27	11	16	18 (17.6)
Neighbourhood 3	0	22	5	32	11	30	16 (16.0)
Neighbourhood 4	0	150	11	80	7	36	18 (6.3)
Zonal Total (%)	3 (0.9)		36 (14.0)		52 (24.2)		
Overall proportion of *Bb*-positive mice	3.3		39.6		57.1		

Supplementary Table S3. Predicted nymphal and tick density by neighbourhoods and zones, based on average marginal effects calculated from models, with all other covariates at their mean value. Density of infected nymphs and infected adults and nymphs combined is determined by the product of the predicted density and the observed infection prevalence (Table 1).

		Nymphal density (nymphs / 100 m^2)	Tick density (ticks / 100 m^2)	Infected nymph density (infected nymphs / 100 m^2)	Infected tick density (infected ticks / 100 m^2)
N1	Residential	0.01	0.02	0.00	0.00
	Interface	0.05	0.40	0.00	0.10
	Woodland	0.17	0.58	0.02	0.13
	All N1	0.09	0.37	0.01	0.09
N2	Residential	0.02	0.04	0.01	0.01
	Interface	0.07	0.80	0.04	0.27
	Woodland	0.24	1.16	0.06	0.39
	All N2	0.12	0.74	0.03	0.25
N3	Residential	0.01	0.01	0.01	0.01
	Interface	0.03	0.18	0.01	0.06
	Woodland	0.11	0.26	0.01	0.06
	All N3	0.06	0.16	0.01	0.04
N4	Residential	0.01	0.01	0.00	0.00
	Interface	0.04	0.25	0.03	0.11
	Woodland	0.14	0.36	0.04	0.13
	All N4	0.07	0.23	0.03	0.09
All	Residential	0.01	0.02	0.01	0.01
	Interface	0.05	0.43	0.01	0.14
	Woodland	0.17	0.62	0.03	0.19

Supplementary Figure S1. Number of ticks of each life stage by month collected, 2020-2021, across all neighbourhoods.

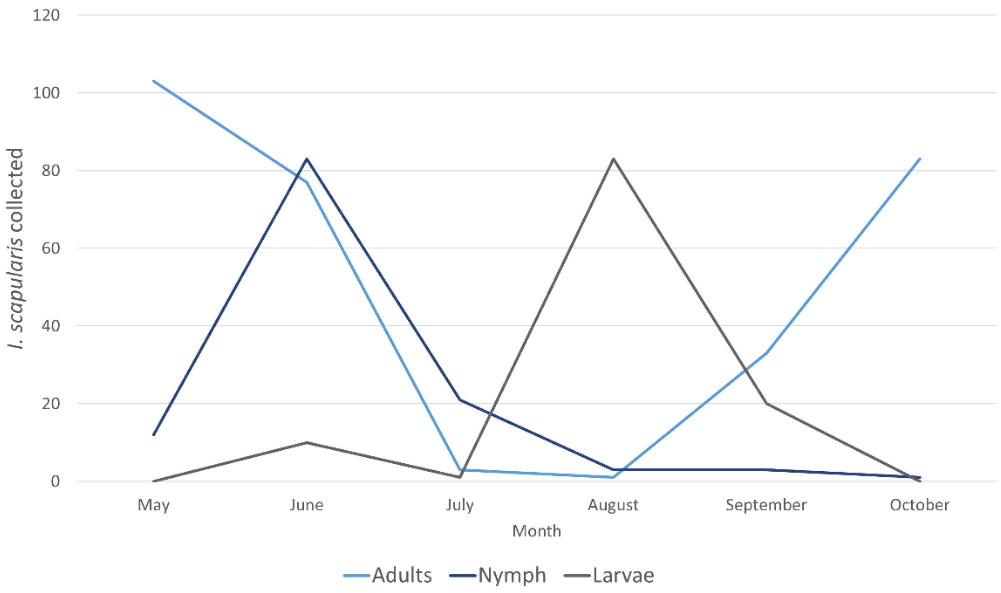

Supplementary Figure S2. Diagnostic residual plots demonstrating negative binomial GLMM fit for the density of nymphal *Ixodes scapularis* ticks.

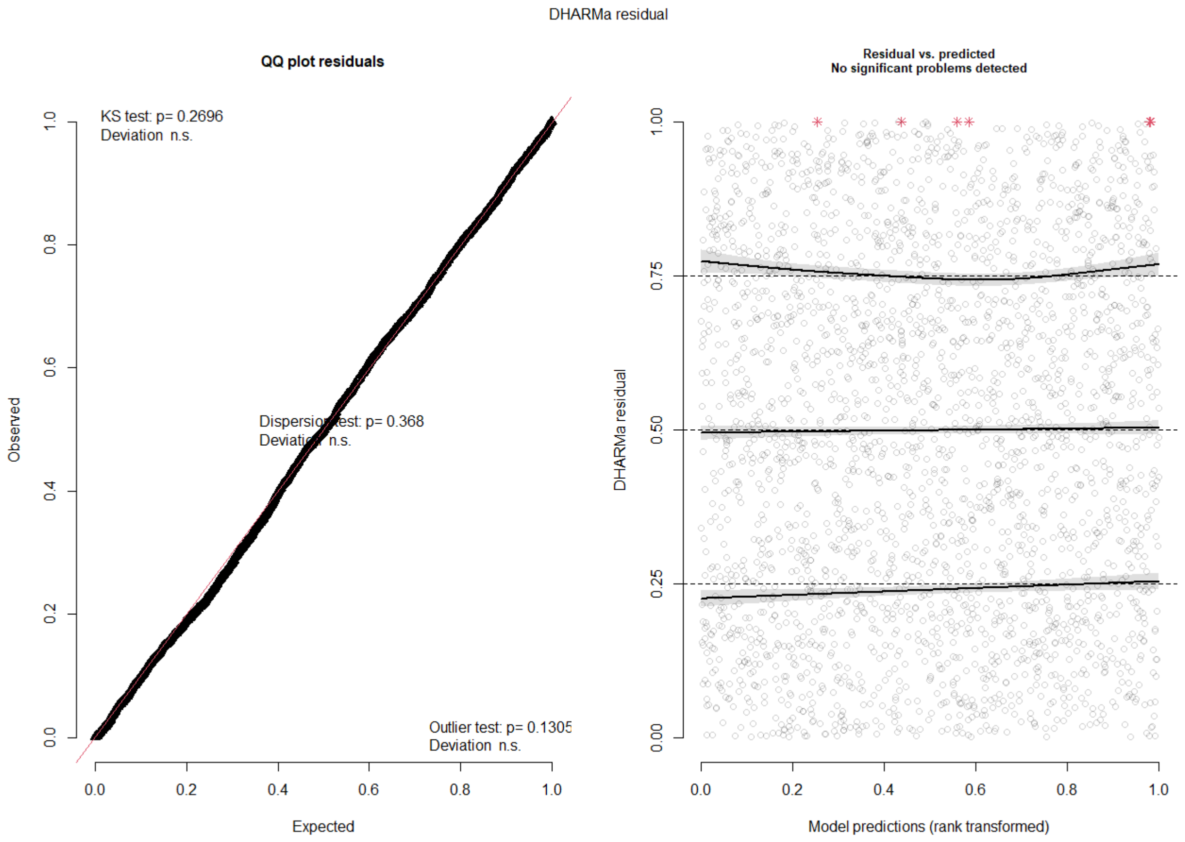

Supplementary Figure S3. Diagnostic residual plots demonstrating Binomial GLMM fit for the *Borrelia burgdorferi* infection prevalence among nymphal *Ixodes scapularis* ticks.

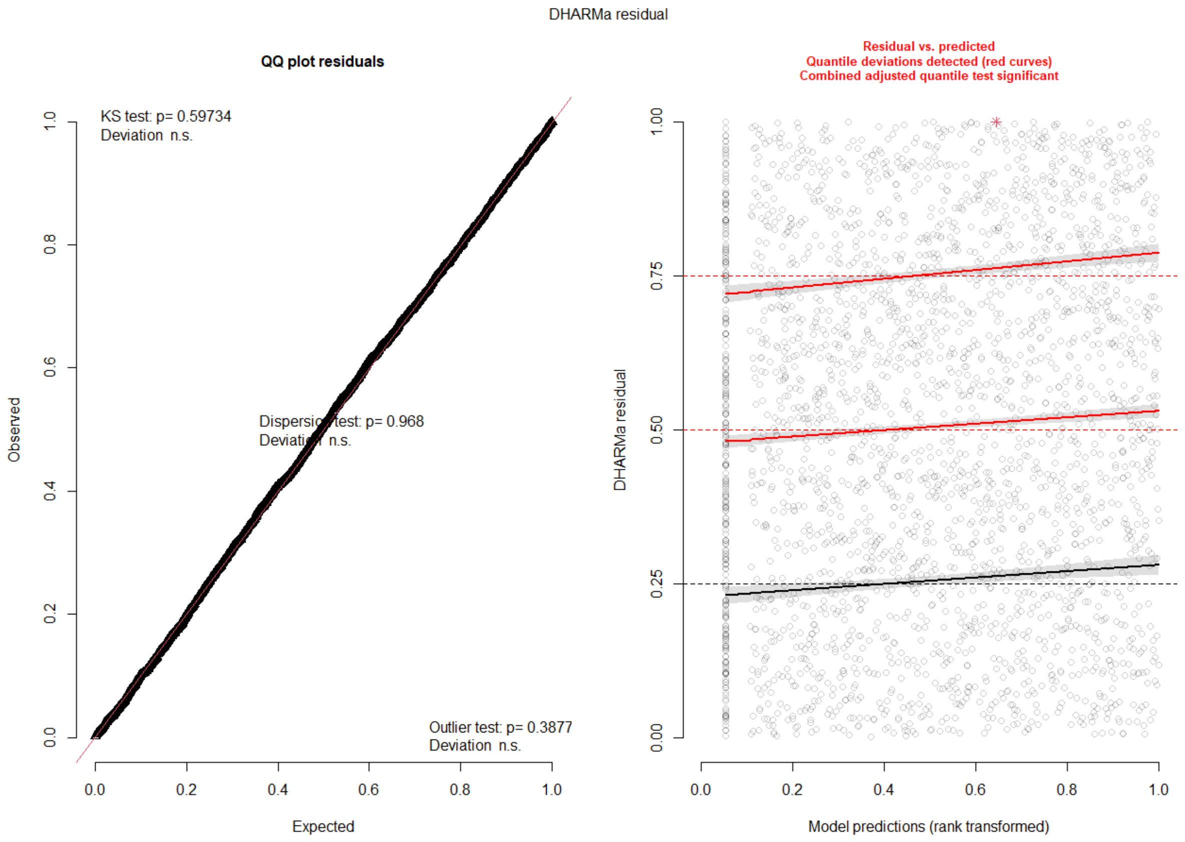

Supplementary Figure S4. Diagnostic residual plots demonstrating negative binomial GLMM fit for the density of nymphal and adult *Ixodes scapularis* ticks.

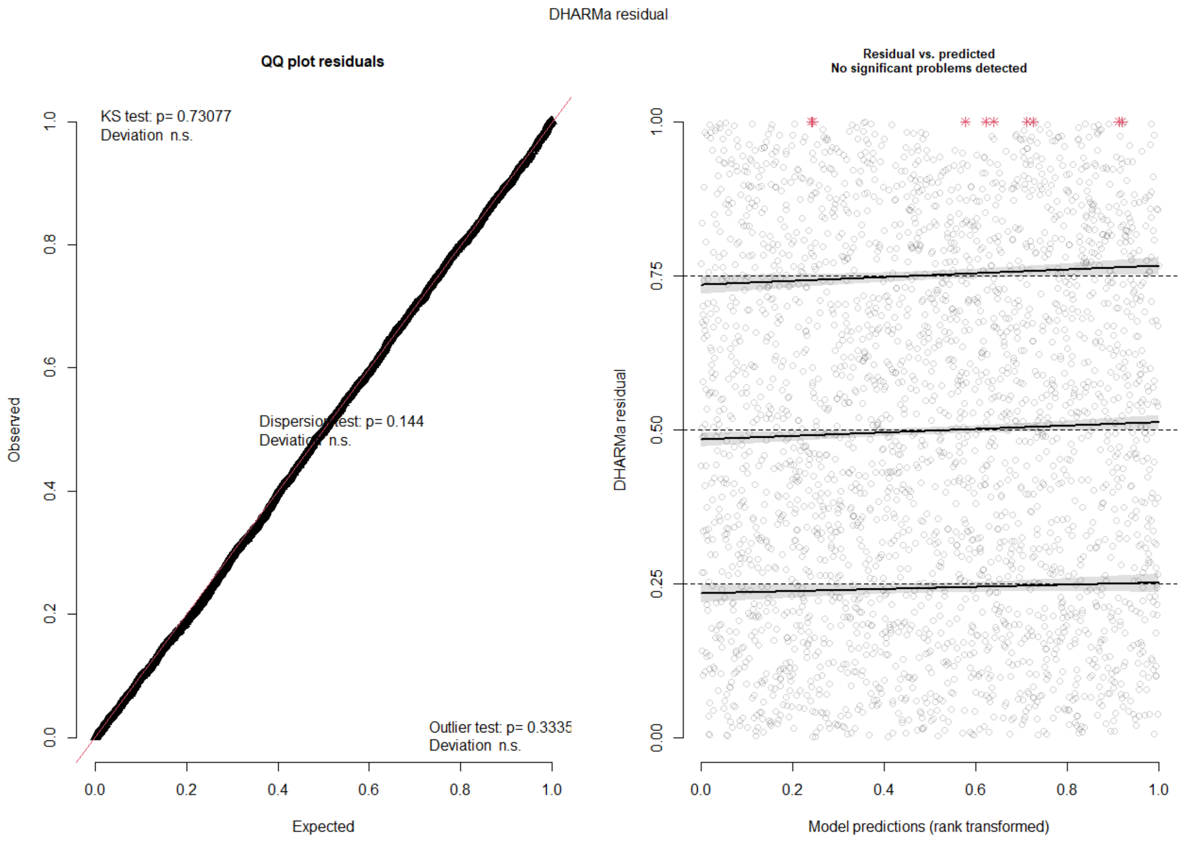

Supplementary Figure S5. Diagnostic residual plots demonstrating Binomial GLMM fit for the *Borrelia burgdorferi* infection prevalence among nymphal and adult *Ixodes scapularis* ticks.

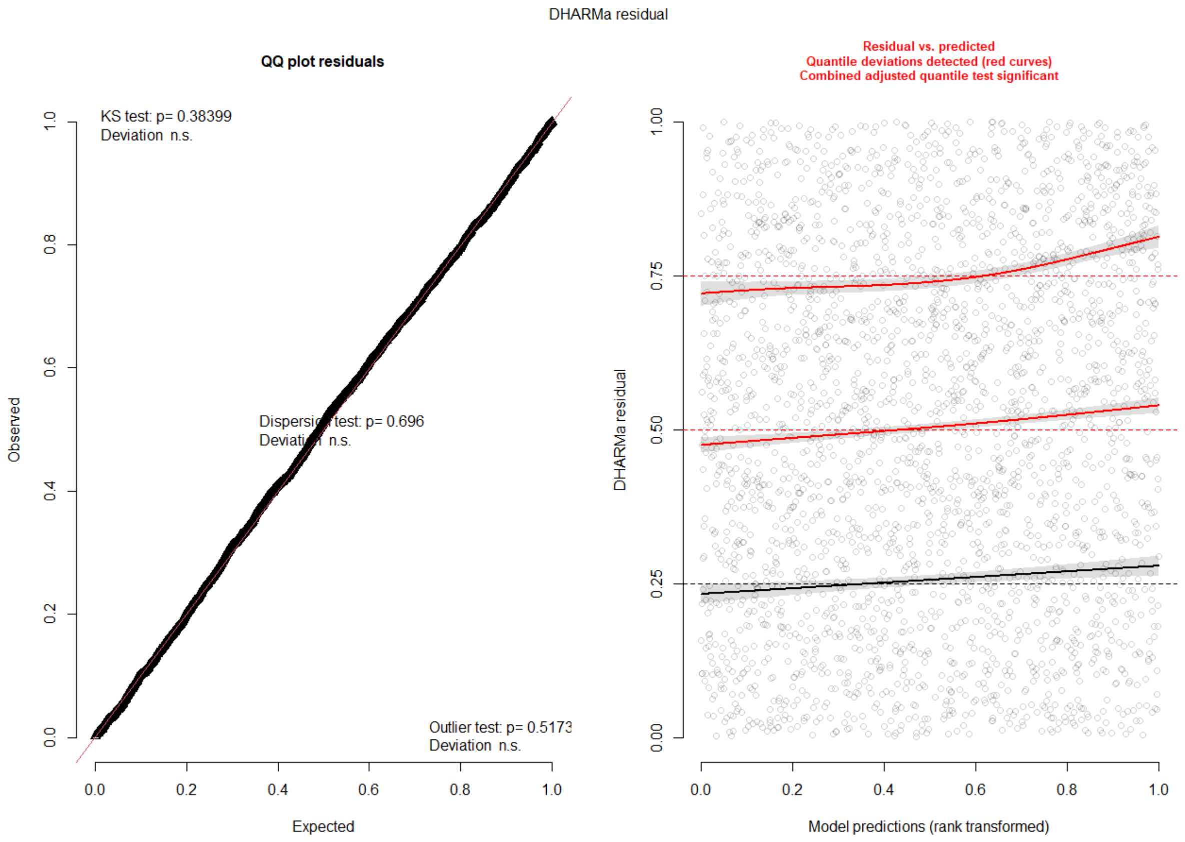

Supplement: Supplementary file 1 — Supplementary Information. [file 41598_2024_64085_MOESM1_ESM.pdf]
